# Monitoring the promoter activity of long noncoding RNAs and stem cell differentiation through knock-in of sgRNA flanked by tRNA in an intron

**DOI:** 10.1038/s41421-021-00272-3

**Published:** 2021-06-15

**Authors:** Yu-Ting Zhao, Yangming Wang

**Affiliations:** 1 Academy for Advanced Interdisciplinary Studies, Peking University, Beijing, China; 2 Institute of Molecular Medicine, College of Future Technology, Peking University, Beijing, China

**Keywords:** Long non-coding RNAs, Embryonic stem cells

Dear Editor,

The majority of mammalian genome is transcribed to RNA transcripts, of which only a very small percentage code for proteins^1^. As a result, thousands of RNAs that do not code for proteins are produced in cells, including microRNAs (miRNAs) and long noncoding RNAs (lncRNAs). These noncoding RNAs exert regulatory functions in various physiological and pathological conditions^2^. In addition, numerous noncoding RNAs are expressed in a tissue- and cell-specific manner^1^. Thus, a reporter that faithfully reflects the expression or activity of noncoding RNAs can provide useful tools not only for uncovering the regulators of noncoding RNAs, but also for tracking cell fate and disease status. Previously we have designed a miRNA inducible CRISPR-Cas9 platform that can serve as a sensor for miRNA activities^3^. However, designing a reporter for long noncoding RNAs has not been easy due to its untranslatable nature and low expression level. Here, we design an sgRNA precursor in an intron (GRIT) strategy that can monitor the promoter activity of lncRNAs (Fig. 1a). Furthermore, we show that GRIT can be used to track differentiation status of stem cells.

**Fig. 1:**
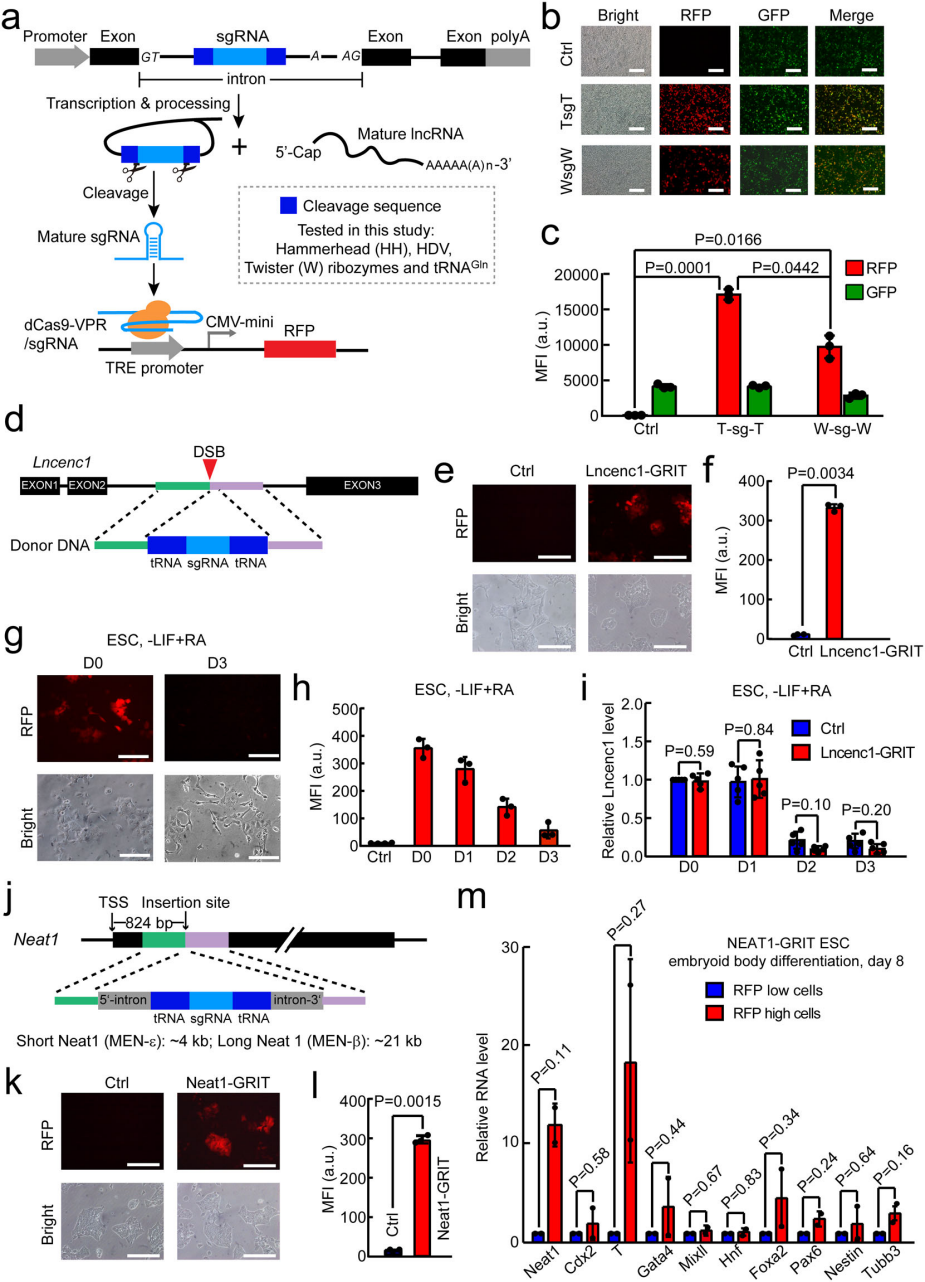
GRIT monitors the promoter activity of lncRNAs and stem cell differentiation. **a** Schematic design of GRIT reporter system. GRIT cassette refers to pre-sgRNAs located in an intron. After the transcription of host gene, removal of flanking RNA cleavage sequences leads to the maturation of sgRNA, which activates the downstream CRISPR-on reporter system. Hammerhead, HDV and Twister ribozyme sequences are in Supplementary Table S1. **b** Representative microscopy images showing RFP and GFP expression in HEK293T transfected with dCas9-VPR, TRE3G-RFP, and GRIT-GFP plasmids. For control, GFP plasmid without any sgRNA cassette in the intron was transfected with dCas9-VPR and TRE3G-RFP plasmids. The schematic for the design of this experiment is shown in Supplementary Fig. S1a. Scale bars, 200 μm. The experiments were repeated three times independently with similar results. TsgT, tRNA-flanked sgRNA. WsgW, Twister ribozyme-flanked sgRNA. **c** Quantification of mean RFP and GFP intensity of **b**. Shown are mean ± SD, *n* = 3 independent experiments. The *P-*value was calculated by one-way ANOVA with two-tailed Tukey’s multiple comparisons test. **d** Schematic of GRIT knock-in strategy for *Lncenc1*. After the establishment of dCas9-VPR and TRE3G-RFP transgenic mouse ESCs, the TsgT element is knocked in the second intron of *Lncenc1* locus through CRISPR-Cpf1-assisted homologous recombination. **e** Representative images showing RFP expression in Lncenc1-GRIT ESCs. Scale bar, 200 μm. **f** Mean RFP intensity of Lncenc1-GRIT ESCs and control ESCs. Shown are mean ± SD, *n* = 3 independent experiments. The *P*-value was calculated using two-tailed unpaired Student’s *t*-test. **g** Representative images showing RFP expression in undifferentiated and differentiated Lncenc1-GRIT ESCs. Scale bar, 200 μm. **h** Quantification of mean RFP intensity during the continuous differentiation process of Lncenc1-GRIT mESCs. Shown are mean ± SD, *n* = 3 independent experiments. **i** RT-qPCR analysis of Lncenc1 expression during the differentiation process of Lncenc1-GRIT and control ESCs. Shown are mean ± SD, *n* = 5 independent experiments. The *P-*value was calculated using two-tailed paired Student’s *t*-test. **j** Schematic of GRIT knock-in strategy for *Neat1* locus. An artificial intron based on the second intron of human *RPL18a* gene containing GRIT elements was knocked into the *Neat1* locus. **k** Representative images showing RFP expression in Neat1-GRIT cells. Scale bar, 200 μm. **l** Mean RFP intensity of Neat1-GRIT ESCs. Shown are mean ± SD, *n* = 3 independent experiments. The *P*-value was calculated using two-tailed unpaired Student’s *t-*test. **m** RT-qPCR analysis of various differentiation markers in RFP high and low cells from day 8 differentiating embryoid bodies of Neat1-GRIT ESCs. EB, embryoid body. Shown are mean ± SD, *n* = 2 independent experiments. The *P-*value was calculated using two-tailed paired Student’s *t*-test. Control ESCs for Lncenc1-GRIT or Neat1-GRIT were ESCs with dCas9-VPR and TRE3G-RFP transgenically integrated but without knock-in of TsgT cassette.

The design of GRIT includes three key elements (Fig. 1a): dCas9-VPR expressed under the control of a constitutively active CAGGS promoter^3^, an RFP gene under the control of a tetracycline-inducible promoter (TRE)^3^, and a transfer RNA^Gln^ (tRNA^Gln^)^4^-flanked sgRNA that is integrated in an endogenous noncoding RNA locus through homologous recombination. To minimize the impact of tRNA-sgRNA knock-in on lncRNAs, we chose genome region that will be expressed as an intron to knock-in tRNA-sgRNAs. In addition, for lncRNA gene without an intron, an artificially designed mini-intron-containing tRNA-sgRNA fusion sequence was knocked in. The tRNA flanking design was chosen based on our observation that tRNA-flanked sgRNA induced higher level of RFP expression when compared to ribozyme-flanked sgRNAs (Fig. 1b, c; Supplementary Fig. S1a-c and Table S1).

We then knocked the tRNA-flanked sgRNA into the second intron of Lncenc1 in mouse embryonic stem cells (ESCs) in which CAGGS-dCas9-VPR and TRE-RFP have been transgenically integrated (Fig. 1d; Supplementary Table S1). Lncenc1 is a lncRNA specifically expressed in mouse ESCs^5^. In ESCs with GRIT successfully integrated (Lncenc1-GRIT ESCs), we observed high level of RFP expression (Fig. 1e, f). In addition, the knock-in of tRNA-sgRNA have little effect on the expression of Lncenc1 and pluripotency genes including Nanog, Oct4 (also known as Pou5f1) and Sox2 (Supplementary Fig. S2a, Tables S2 and S3). Importantly, the transcription activity of Lncenc1 locus was found not affected based on qPCR analysis of precursor RNA of Lncenc1 (Supplementary Fig. S2a).

Lncenc1 is downregulated during ESC differentiation^5^. To check whether GRIT can report the expression of Lncenc1 during ESC differentiation, we induced differentiation of Lncenc1-GRIT ESCs with all-trans retinoid acids (ATRA) and measured RFP expression during differentiation process. Interestingly, RFP was significantly decreased upon ATRA induced differentiation (Fig. 1g, h; Supplementary Fig. S2b). More importantly, RFP level was highly correlated to the RNA level of Lncenc1 (Supplementary Fig. S2c). Furthermore, by comparing the expression of Lncenc1 and Oct4 during the differentiation process of control and Lncenc1-GRIT ESCs (Fig. 1i; Supplementary Fig. S2d), we concluded that knock-in of tRNA-flanked sgRNA did not impact the differentiation potential of mouse ESCs. To check whether RFP level may reflect the stages of differentiation, we sorted out high and low RFP populations in day 2 differentiated Lncenc1-GRIT cells and analyzed the expression of pluripotency genes (Supplementary Fig. S3a). Interestingly, Oct4, Nanog, and Sox2 were indeed higher in RFP high cells than in RFP low cells (Supplementary Fig. S3b). These results demonstrate the potential of GRIT to report the promoter activity of lncRNAs and as an indicator to monitor the differentiation status of stem cells.

NEAT1 is a lncRNA serving as a structural organizer of paraspeckle and has been shown to play important roles from gene regulation to cancer progression^6^. In addition, NEAT1 is an intronless gene. We constructed NEAT1-GRIT ESCs by inserting a mini-intron containing tRNA-flanked sgRNA (Fig. 1j). As expected, RFP was significantly induced in NEAT1-GRIT ESCs (Fig. 1k, l). In addition, the insertion of mini-intron did not affect the expression of NEAT1 RNA and pluripotency genes including Oct4, Nanog, Sox2, and Klf4 (Supplementary Fig. S4a). We then performed embryoid body differentiation of NEAT1-GRIT ESCs and sorted out RFP high cells at day 8 (Supplementary Fig. S4b). As expected, qRT-PCR analysis showed that RFP high cells express higher level of NEAT1 (Fig. 1m). In addition, mesoderm marker T brachyurary was highly upregulated in RFP high cells (Fig. 1m), indicating that high NEAT1 expression may mark certain cell lineages from mesoderm. Finally, we made a GRIT reporter for H19 lncRNA to drive TRE-GFP expression (Supplementary Fig. S5a). Consistently, GFP expression was highly induced in H19-GRIT ESCs (Supplementary Fig. S5b) and the knock-in of GRIT did not affect the expression of H19 lncRNA or pluripotency genes (Supplementary Fig. S5c). Together, these data demonstrate the generality of GRIT to serve as a reporter for monitoring the promoter activity of noncoding RNAs.

In summary, we constructed a lncRNA reporter GRIT with CRISPR-on system by insertion of a tRNA-flanked sgRNA in endogenous lncRNA loci. We showed that GRIT is useful for tracking ESC differentiation and labelling specific cell lineages. A recent study from Gao et al. reported a similar design named as Ents^7^. Ents uses ScFV-P65-HSF1 instead of VPR to activate gene expression and optimized mini-CMV-mCherry as a reporter. Different from traditional methods to report promoter activity by knocking in a protein such as GFP or luciferase, both GRIT and Ents strategies knock-in a smaller DNA fragment that will not change the noncoding status of lncRNAs. In Ents strategy, the authors knocked in the sgRNA cassette downstream of the polyA site. Here we knocked in the sgRNA cassette in the middle of ncRNA sequences. While the Ents strategy may have less impact than the GRIT strategy on the expression or function of monitored genes, the faithfulness of Ents in monitoring the promoter activity could be reduced in certain cases since the cleavage at the polyA site may affect the stability of downstream transcript. In the case of *Lncenc1* in mouse ESCs, Ents strategy achieved ~3-fold induction of mCherry expression, while the GRIT strategy in this study achieved ~35-fold induction of RFP expression. However, the exact effectiveness of two strategies can only be concluded when both strategies are compared in the same system side by side. In addition, for both Ents and GRIT, the expression of dCas9 or associated proteins could affect their faithfulness in monitoring the promoter activity. This issue may be addressed by knocking the expression cassette of dCas9 and associated proteins into constitutively active gene loci. Finally, when sgRNAs are designed to target endogenous DNA locus, GRIT may be utilized to edit or manipulate the expression of endogenous genes. We expect that GRIT will be applicable in uncovering molecular mechanisms regulating the transcription of lncRNAs, tracking cell fate switch during differentiation, reprogramming or disease progression and integrating the promoter activity for synthetic biology applications.

## Supplementary information

Supplementary information

Supplementary Tables S1-2

Supplementary Table S3
