# Supplementary material for: Monitoring the promoter activity of long noncoding RNAs and stem cell differentiation through knock-in of sgRNA flanked by tRNA in an intron
Source: Cell Discov. 2021 Jun 15;7:45. doi: 10.1038/s41421-021-00272-3 (PMC8203696; doi:10.1038/s41421-021-00272-3)
Supplement: Supplementary file 1 — Supplementary information [file 41421_2021_272_MOESM1_ESM.pdf]

Supplementary Information

Monitoring the promoter activity of long noncoding RNAs and stem cell differentiation through knock-in of sgRNA flanked by tRNA in an intron

Authors: Yu-Ting Zhao[1], Yangming Wang[2,*]

Affiliations:

1 Academy for Advanced Interdisciplinary Studies, Peking University, Beijing, China

2 Institute of Molecular Medicine, College of Future Technology, Peking University, Beijing, China

*Correspondence: Yangming Wang (yangming.wang@pku.edu.cn)

This file contains:

Supplementary Figures S1-5 with Figure Legends

Legends for Supplementary Tables S1-3

Supplementary Materials and Methods

Supplementary Figure S1

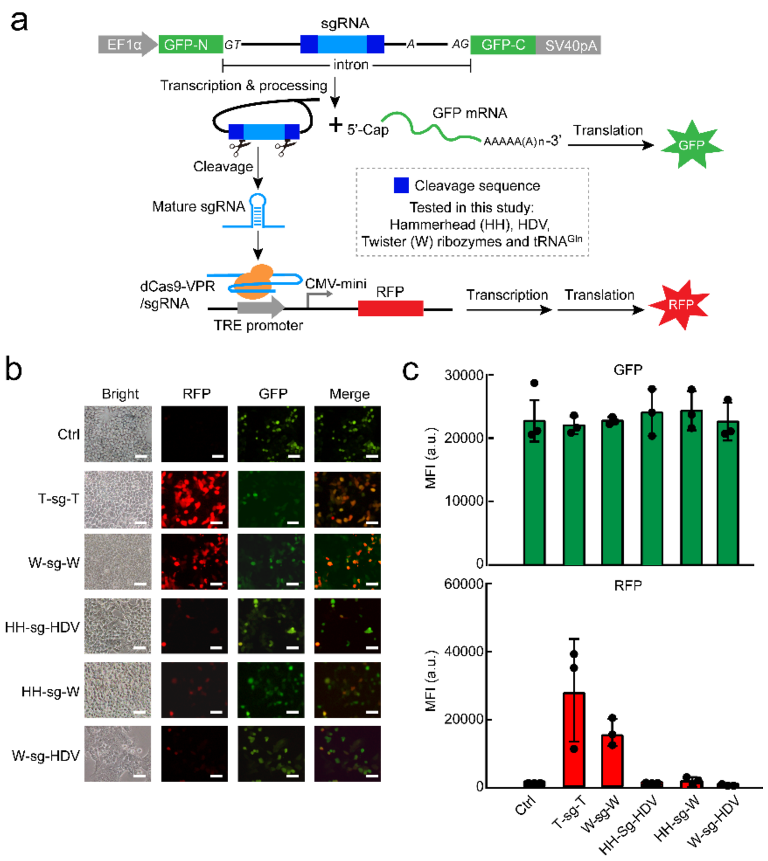

Supplementary Figure S1. tRNA and twister ribozyme sequences flanking sgRNAs in GRIT lead to high RFP induction.

a) Schematic representation of GRIT in an exogenous GFP gene. The pre-sgRNA in an intron cassette is inserted in GFP sequence driven by EF1α promoter. The intron was derived from the second intron of human *RPL18a* gene.

b) Representative microscopy images showing the expression of RFP and GFP for tested GRIT cassettes in HEK293T. Scale bar, 50µm. For control, GFP plasmid without any sgRNA cassette in the intron was transfected with dCas9-VPR and TRE3G-RFP plasmids.

c) RFP and GFP intensity of GRIT cassettes overexpressed in HEK293T. Shown are mean ± SD, n = 3 independent experiments.

Supplementary Figure S2

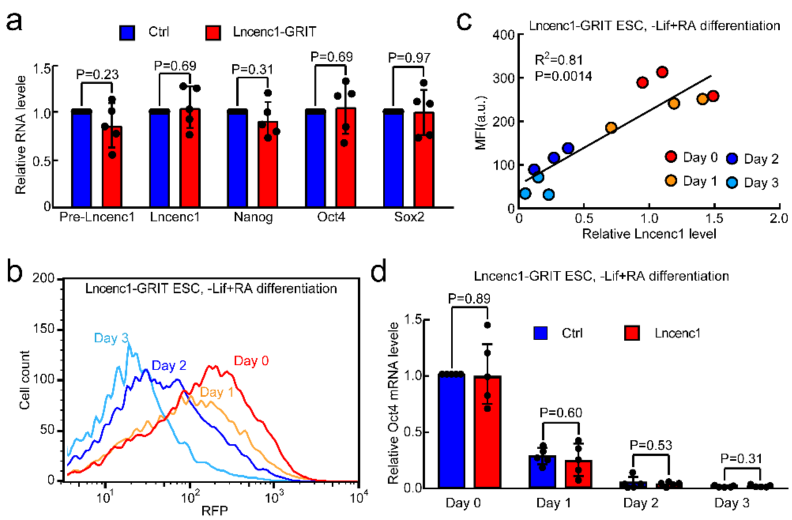

Supplementary Figure S2. GRIT has little impact on the expression of Lncenc1 or the differentiation process of ESCs.

a) RT-qPCR analysis of control and Lncenc1-GRIT ESCs. Control ESCs refer to embryonic stem cell line stably expressed dCas9-VPR and TRE3G-RFP with no GRIT knocked in. β-actin mRNA was used as a control. Data were normalized to control ESCs. Shown are mean ± SD, n = 5 independent experiments. The *p*-value was calculated by two-tailed paired Student’s *t* test.

b) Representative flow cytometry analysis of undifferentiated and ATRA differentiated Lncenc1-GRIT cells.

c) Linear regression analysis between Lncenc1-GRIT RFP level and Lncenc1 RNA level during ESC differentiation. R^2, the square of Pearson correlation coefficient.

d) RT-qPCR analysis of Oct4 during differentiation process of control and Lncenc1-GRIT ESCs. β-actin mRNA was used as a control. Data were normalized to control ESCs. Shown are mean ± SD, n = 5 independent experiments. The p-value was calculated using two-tailed paired Student’s *t* test.

Control ESCs for Lncenc1-GRIT were ESCs with dCas9-VPR and TRE3G-RFP transgenically integrated but without knockin of TsgT cassette.

Supplementary Figure S3

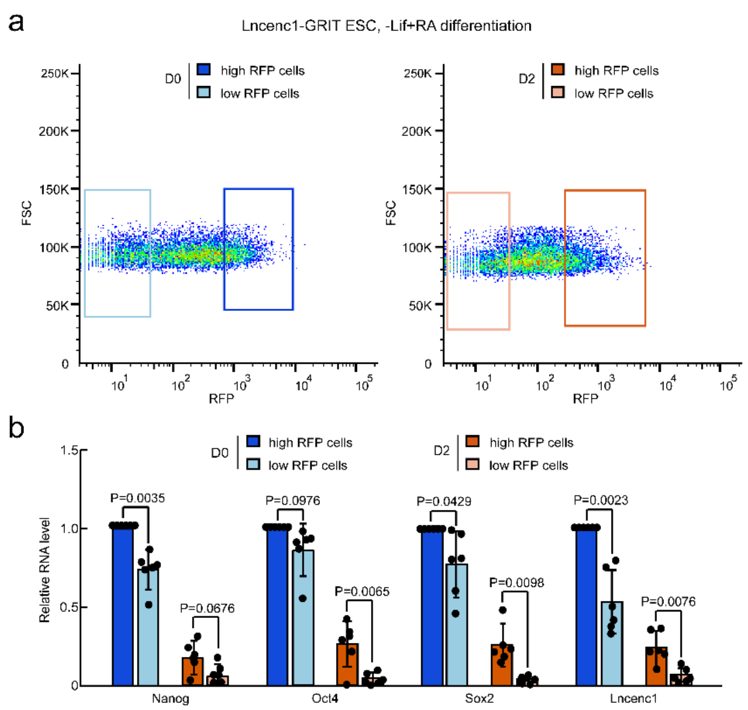

Supplementary Figure S3. Lncenc1-GRIT can track the differentiation status of ESCs.

a) Representative flow cytometry scatter plot and sorting gates of RFP high and low cells for undifferentiated and differentiated Lncenc1-GRIT ESCs.

b) RT-qPCR analysis of RFP high and low undifferentiated and differentiated Lncenc1-GRIT ESCs. Data were normalized to β-actin and then undifferentiated high RFP intensity ESCs. Shown are mean ± SD, n = 6 independent experiments. The p-value was calculated using two-tailed paired Student’s *t* test.

Supplementary Figure S4

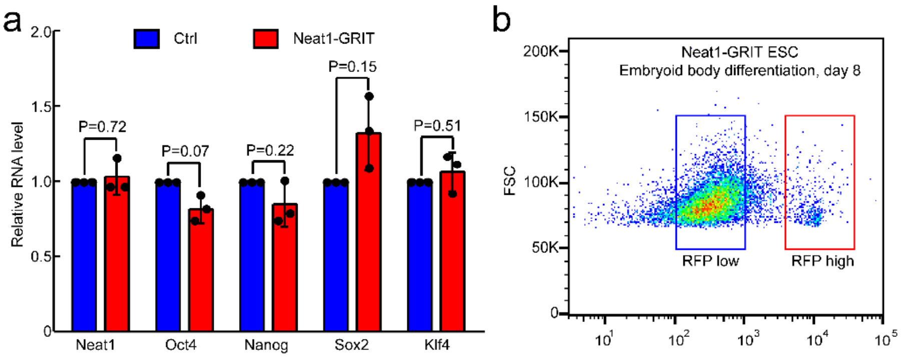

Supplementary Figure S4. GRIT can track the expression of Neat1 in mouse ESCs.

a) RT-qPCR analysis of control and Neat1-GRIT ESCs. Control ESCs are dCas9-VPR and TRE3G-RFP transgenic ESCs without knockin of TsgT cassette. β-actin mRNA was used as a control. Data were normalized to control ESCs. Shown are mean ± SD, n = 3 independent experiments. The p-value was calculated using two-tailed paired Student's *t* test.

b) Representative flow cytometry scatter plot and sorting gates of RFP high and low cells from day 8 embryoid bodies of Neat1-GRIT ESCs.

Supplementary Figure S5

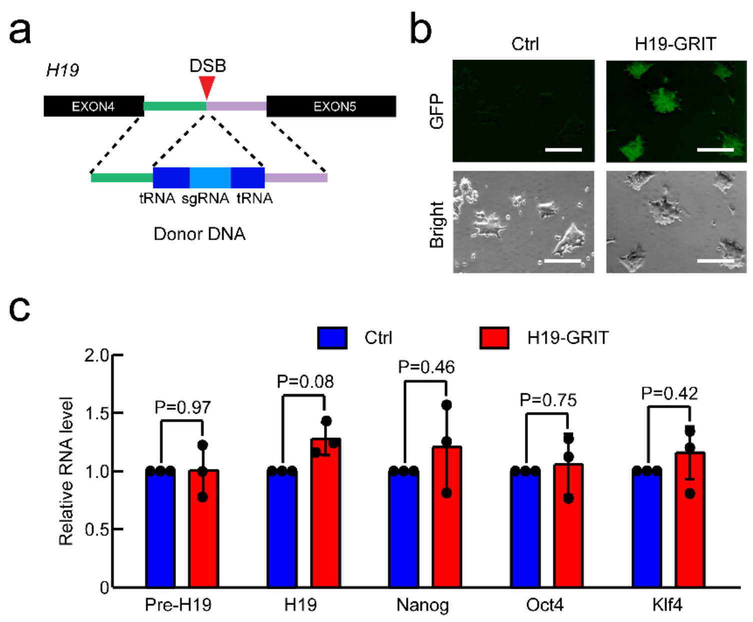

Supplementary Figure S5. Knockin of GRIT in H19 does not affect the expression of H19 or pluripotency genes.

a) GRIT knock-in strategy for H19. After the establishment of dCas9-VPR and TRE3G-GFP transgenic mouse ESCs, the TsgT element is knocked in the fourth intron of H19 locus through CRISPR-Cpf1-assisted homologous recombination.

b) Representative images showing GFP expression in H19-GRIT ESCs. Scale bar, 200 μm.

c) RT-qPCR analysis of control and H19-GRIT ESCs. Control ESCs refer to ESCs stably expressing dCas9-VPR and TRE3G-GFP with no TsgT element knocked in. β-actin mRNA was used as a control. Data were normalized to control ESCs. Shown are mean ± SD, n = 3 independent experiments. The p-value was calculated by two-tailed paired Student's *t* test.

Control ESCs are dCas9-VPR and TRE3G-GFP transgenic ESCs without knockin of TsgT cassette.

Legends for Supplementary Tables S1-3

Supplementary Table S1. The oligos and primers for constructing GRIT cassettes.

Supplementary Table S2. Sequences for real time qPCR primers.

Supplementary Table S3. Source data for figures 1c, 1f, 1h, 1i, 1l, 1m and S1c, S2a, S2c, S2d, S3b, S4a and S5c.

Supplementary Materials and Methods

Plasmid and vector construction

The dCas9–VPR expression was driven by a CAGGS promoter in a piggyBac vector containing a hygromycin resistance gene driven by a PGK promoter. RFP was cloned downstream of an inducible TRE3G promoter in a piggyBac vector containing a blasticidin resistance gene. The TRE3G promoter contains seven repeats of sgRNA-binding site (5'-TACGTTCTCTATCACTGATAGGG-3'). For experiments testing different flank sequences, the pre-sgRNAs were placed between the 312th and 313th position of the second intron of human *RPL18a* gene. Next, the pre-sgRNAs in intron were embedded into GFP coding sequence. The two parts of the intron were cloned from human genomic DNA separately. The N terminal and C terminal parts of GFP were cloned by PCR. All the PCR products were generated by Phanta® HS Super-Fidelity DNA Polymerase (Vazyme, Cat. #P502-d) and purified by a FastPure Gel DNA Extraction (Vazyme, Cat. #DC301).The five PCR fragments (GFP-N, intron-5' part, pre-sgRNAs, intron-3' part, GFP-C) were assembled by recombinase (Vazyme, Cat. # C113). Primers for cloning these constructs are listed in Supplementary Table S1. Sequences for Hammerhead, HDV and Twister ribozymes are from references 1 and 2.

The donor templates, consisting of 5' arm and 3' arm for knock-in were cloned from mouse genomic DNA. For Lncenc1-GRIT, the primers for homology arms cloning introduced restriction endonuclease site PacI and PmeI. Meanwhile, the PCR products

of T-sg-T were digested with PacI and PmeI (NEB, Cat. # R0547 and Cat. # R0560) and purified. The PacI and PmeI digested homology arms and T-sg-T PCR product were then ligated using T4 ligase (Thermofisher, Cat. # EL0012) to construct Lncenc1-GRIT knock in template. For Neat1-GRIT, the left and right homology arms and T-sg-T in *RPL18a* intron were separately amplified by PCR. The three parts were then assembled together by overlapping PCR and cloned into pEASY Blunt Simple vector. The primers for cloning homology arms are listed in Supplementary Table S1.

Cell culture and construct of reporter cell lines

HEK293T cells were cultured at 37 °C under 5% CO_2 in high glucose Dulbecco's Modified Eagle's Medium (DMEM, Hyclone, Cat. #SH30243.01) supplemented with 10% FBS (PANSera, Cat. #2602−P130707). Embryonic stem cells were grown on gelatin-coated dish in KnockOut^{TM} DMEM (Gibco, Cat. # 10829081) supplemented with 15% FBS (Hyclone, Cat. # SH3007103), 1,000 U/ml mouse leukemia inhibitory factor (1,000 U/ml), 0.1 mM non-essential amino acids (Gibco, Cat. # 11140050), 1 mM L-glutamine (Gibco, Cat. # 25030081), 0.1 mM β-mercaptoethanol, and penicillin (100 U/ml) and streptomycin (100 μg/ml).

To establish GRIT reporter ESCs, we first generated mouse V6.5 ESCs in which dCas9-VPR and TRE3G-RFP (or TRE3G-GFP) were stably integrated. dCas9-VPR and TRE3G-RFP (or TRE3G-GFP) plasmids were co-transfected along with PBase expression plasmids using Polyplus Transfection (Jetprime, Cat. # PT-114-75) reagent. After transfection, cells were treated with 10 μg/ml Blasticidin S (Gibco) and 150 μg/ml

Hygromycin (Roche) for 4 days. Then monoclonal cell line was selected and expanded for further experiments. The GRIT cassettes were then knocked in the host gene locus assisted with CRISPR/AsCpf1 system through HDR (homology-directed repair) strategy. For Lncenc1-GRIT ESCs, T-sg-T cassette was knocked into the second intron of Lncenc1. The target site was CTCCTACCTCTCAAATAAATAGTT, with TTTA PAM (protospacer adjacent motif) sequences. For Neat1-GRIT ESCs， T-sg-T in intron cassette was knocked in Neat1 locus, and the target site was AGTGACCCCTTAACCTCAGAGTGA, with TTTA PAM sequence. For H19-GRIT ESCs, T-sg-T cassette was knocked into the fourth intron of H19. The target site is GAAGCTTGCCAAGCCCACTCCCCA, with TTTG PAM sequences. The crRNAs for Cpf1 were designed by CHOPCHOP (https://chopchop.cbu.uib.no/).

For monolayer retinoid acid differentiation, 50,000 ESCs per well were seeded in ESC media in 6-well plates. After 24hr, the medium was changed to differentiation media without LIF and in the presence of 100 nM all-trans retinoid acid. For embryoid body differentiation assay, 200,000 ESCs per well were plated in suspension in 6-well Ultra-Low attachment plates (Corning, Cat. # 3471) in basic medium with 10% FBS and without LIF. Media were changed every other day until further experiments.

RNA extraction and RT-qPCR

Total RNA was extracted from cells with Trizol reagent (Invitrogen, Cat. # 15596026). For quantitative PCR with reverse transcription (RT-qPCR) analysis, cDNA were typically obtained from 500 ng total RNA using HiScript II Q RT SuperMix for qPCR

(Vazyme, Cat. # R223). qPCR assay was carried out with AceQ qPCR SYBR Green Mater Mix reagent (Vazyme, Cat. # Q141) in 96-well plates on StepOne Plus Real-Time PCR System (Applied Biosystems) with standard protocols. Primers for qPCR are listed in Supplementary Table S2.

Fluorescence activated cell sorting and flow cytometry analysis

Cells were first dissociated with 0.1% Trypsin and then collected in PBS containing 2% FBS. Cell sorting was performed on BD FACS Aria III. For analysis of flow-cytometry in Figure 1c and S1c, HEK293T cells were collected at 48hrs after transfection. Flow cytometry for quantifying population and fluorescence intensity was performed by BD LSR Fortessa SORP. Data were analyzed using FlowJo software.

Quantification and statistical analysis

The number of independent experimental replications, the definition of center and precisions measures are reported in the figure legends (n, mean ± SD). $p<0.05$ is generally considered as statistically significant. Statistical analyses were performed using the GraphPad Prism v6 software. Statistical significance was assessed by two-tailed Student's *t*-test. For multiple comparison, the p-value was calculated by one-way ANOVA followed with Tukey's multiple comparisons test.

Data and materials availability

All data generated or analyzed during this study are included in the manuscript and its supplementary information files. Source data in figures 1c, 1f, 1h, 1i, 1l, 1m and supplementary figures S1c, S2a, S2c, S2d, S3b, S4a and S5c are provided in

Supplementary Table S3. Original data used and/or analyzed during the current study are available from the corresponding author on reasonable request.

References

1 Yoshioka, S., Fujii, W., Ogawa, T., Sugiura, K. & Naito, K. Development of a mono-promoter-driven CRISPR/Cas9 system in mammalian cells. *Scientific reports* **5**, 18341, doi:10.1038/srep18341 (2015).

2 Litke, J. L. & Jaffrey, S. R. Highly efficient expression of circular RNA aptamers in cells using autocatalytic transcripts. *Nature biotechnology* **37**, 667-675, doi:10.1038/s41587-019-0090-6 (2019).
